# The TSPY gene and the loss of Y chromosome predisposition to cancers

**DOI:** 10.1186/s13578-026-01614-y

**Published:** 2026-06-26

**Authors:** Yun-Fai Chris Lau

**Affiliations:** Department of Medicine, San Francisco VA Health Care System, VA Medical Center, 111C5, University of California, San Francisco, 4150 Clement Street, 94121 San Francisco, CA USA

## Abstract

Mosaic loss of Y chromosome is a common genetic phenomenon in elderly men. It predisposes affected individuals to cancers, suggesting that genes on this male-specific chromosome contribute to the well-being of men. Currently, the putative gene(s) responsible for such cancer predisposition when lost is/are currently unknown. A recent study identified the testis-specific protein Y-encoded (TSPY) gene possesses significant immunogenicity, capable of eliciting significant immune responses against and eliminating positive tumor cells in a mouse model of liver cancer. TSPY is a male-specific cancer-testis antigen expressed in a wide variety of cancers but not normal somatic cells. It is an ampliconic gene constituting a majority (~ 42%) of all protein-coding genes on the human Y chromosome. TSPY is hypothesized to be a guardian gene for man, protecting man from cancer development. Its high copy-number of conserved functional units ensures that it is intrinsically activated in early/progenitor cancer cells, thereby eliciting robust immune responses, eliminating early tumor progenitor cells and preempting oncogenic progression and tumor development. Loss of TSPY gene cluster together with the loss of the entire Y chromosome in oncogenic progenitor/early tumor cells provide a favorable environment fostering oncogenic development, thereby predisposing elderly men to cancers.

**Supplementary Information:**

The online version contains supplementary material available at 10.1186/s13578-026-01614-y.

## Background

### Loss of Y chromosome and cancer predisposition

Mosaic loss of the Y chromosome (mosaic LOY) is a commonly observed phenomenon in elderly men. The severity increases with age and is linked to significant predisposition to various cancers, including prostate, bladder, throat, stomach cancers and leukemia, among others, and is an indicator of poor prognosis and shorter survival for the patients [1]. Further studies suggest that that mosaic LOY is also linked to susceptibility of a variety of illnesses, such as Alzheimer’s disease, schizophrenia, macular degeneration and viral infections [2]. Thus, the Y chromosome, and the genes thereof, participates in the maintenance of the homeostasis of man’s health.

LOY is usually studied in readily available tissues, such as peripheral blood and cancer tissues [1, 3, 4]. Initial studies on the nucleated white blood cells had first established the cancer predisposition link to mosaic LOY among elderly men [1]. It suggests potential genome instability as men age and likely to be extended to other susceptible tissues [5, 6]. Recent studies of large numbers of different types of cancer tissues suggested that LOY could be attributed to two likely roles in cancers, i.e. (a) driver mode propelling high cancer incidence, metastasis and worse outcomes and (b) passenger (passive) LOY as results of unstable genome and mutation of tumor suppressors, such as TP53 [4]. Since nucleated white blood cells are mostly lymphocytes, i.e. immune cells, recent studies have further identified LOY in T cells to play key roles in oncogenic susceptibility [7, 8]. A detailed study of LOY in bladder cancer showed that it occurs in 10–40% of male patients and the Y-negative tumors promote striking dysfunction or exhaustion of CD8+ cytotoxic T cells in the tumor microenvironment, leading to evasion of adaptive immunity and increased patient mortality [9]. Thus, LOY in cancer cells is associated with dysfunctions of the immunosurveillance of cancer. The important question is what on the human Y chromosome that could mediate immunosurveillance and when it/they is/are lost together with the entire Y chromosome promotes immune dysfunctions impairing cancer immunosurveillance.

### The TSPY gene on the Y chromosome

The Y chromosome is the smallest and the most gene-poor chromosome in the human genome [10]. Recent complete telomere-to-telomere (T2T) sequencing of a human Y chromosome identified 106 protein-coding genes on this chromosome, 103 of which have been annotated (Supplementary Table S1) [11]. It harbors about 44 gene/gene families, 17 and 4 single-copy genes are located on the pseudoautosomal region 1 and 2 (PAR1 and PAR2) at the telomere of the short and long arm respectively [12, 13]. Most of the PAR genes have identical genes on the X chromosome and are postulated to serve the same functions. The male-specific Y (MSY) region in the middle harbors about 23 genes/gene families, involved in sex determination (i.e. sex-determining region Y or SRY) and spermatogenesis, and other male functions [11]. Most genes on the MSY region possess counterparts on the X chromosome with various degrees of homology and could possess similar or different functions, such as the testis-specific protein Y-encoded (TSPY) and the testis-specific protein Y-encoded homolog on the X (TSPX) genes [14]. Significantly, there are nine ampliconic gene families with multiple functional members and pseudogenes. They constitute 68 (62%) of the protein-coding genes of the Y chromosome. Importantly, there are 66 copies of TSPY gene, of which 21 are un-transcribed pseudogenes and 45 are protein-coding and highly conserved genes. They represent 42.5% of the total protein-coding genes on the Y chromosome. The 2.8-kb structural gene are embedded in highly conserved 20.3-kb units in a head-to-tail repeat pattern on the plus strand towards the centromere, encompassing close to 1 MB of Y-DNA [11, 15]. One separate unit, TSPY2, lies 385-kb downstream from the main cluster on the minus strand. The exact functional significance, if any, of such peculiar genomic arrangement of highly conserved TSPY repeats is currently unclear. Sequencing of additional 43 human Y chromosomes suggest that the TSPY copy-number could vary from 29 to 66 [11, 16]. These observations raise an important question on why there are so many copies of highly conserved functional TSPY gene on the human Y chromosome.

The TSPY was the third gene isolated from the human Y chromosome in late 1980s and early 1990s [15, 17]. It is a tandemly repeated gene mapped to the critical region on the short arm harboring the gonadoblastoma locus on the Y chromosome (GBY) [18], the only oncogenic locus on this male-specific chromosome. Early studies showed that TSPY is evolutionarily conserved, except the laboratory mouse harboring a nonfunctional pseudogene on its Y chromosome [19]. It is exclusively expressed in the germ cells within the seminiferous tubules of the testis [20]. Importantly, it is aberrantly expressed in various types of cancers, particularly gonadoblastoma, testicular germ cell tumors, prostate cancer and hepatocellular carcinoma (HCC) [21]. Additional studies showed that its oncogenic expression is more widespread, including lung adenocarcinoma, melanoma, head and neck carcinoma, bladder and kidney cancers, Table 1, and at low levels in numerous other somatic cancers, such as glioblastoma/glioma and colon cancer [22]. Early studies demonstrated that TSPY binds cyclin B and stimulates the cyclin B-CDK1 kinase activities, thereby exacerbating the G_2_/M transition in the cell cycle [23, 24], which not only accelerates cell proliferation but also promotes genome instability as mutations/DNA defects during the synthetic (S) phase might not be repaired properly in an abbreviated G_2_/M phase. Its potential oncogenic functions suggest that it is the putative gene for the GBY locus, predisposing dysfunctional germ cells to oncogenesis. Further, the TSPY stimulating effects on cyclin B-CDK1 could be essential for spermatogonial replication and two consecutive rounds of meiotic divisions in the testis [25]. Thus, the TSPY high copy-number could be related to its essential spermatogenic functions. Indeed, mutation and/or copy number variation of TSPY have been linked to variation of male fertility and infertility [26–28].

**Table 1:** Percentage of TSPY positivity in male reproductive and somatic cancers

Cancer type	Percentage of TSPY positivity in male cases	References
Gonadoblastoma	~ 100	[29, 30]
Testicular Germ Cell Tumors	~ 70–90	[30–32]
Prostate Cancer	~ 60–80	[33, 34]
Hepatocellular Carcinoma	33	[22, 35, 36]
Lung Adenocarcinoma	17	[22]
Mesothelioma	14	*
Melanoma	12	*
Head and Neck Carcinoma	11	[22]
Bladder Cancer	10	[22]
Kidney Cancer	6	[22]
Sarcoma	4	*
Rectum adenocarcinoma	2	*

*Unpublished observations from Dr. Tatsuo Kido, personal communication

TSPY expression and cell cycle accelerating effects were postulated to contribute to numerous positive cancers, thereby exerting male-specific effects on oncogenesis [21]. Further, TSPY is a co-activator of the androgen receptor (AR) and its constitutively active variants, such as AR-V7, capable of binding and exacerbating the AR/AR-V transactivation of responsive genes. TSPY could have a positive effect(s) on AR-related male-specific cancers, such as prostate cancer, and other male-biased somatic cancers, such as HCC [37, 38]. Thus, TSPY is a Y-located proto-oncogene contributing to male-specific and/or male-biased oncogenesis. A recent study from our laboratory [39], however, clearly suggests that TSPY could serve a separate and contrasting function possibly related to its high copy-number on the human Y chromosome.

## Hypothesis

### TSPY contribution to loss of Y chromosome and cancer predisposition

**Fig. 1:**
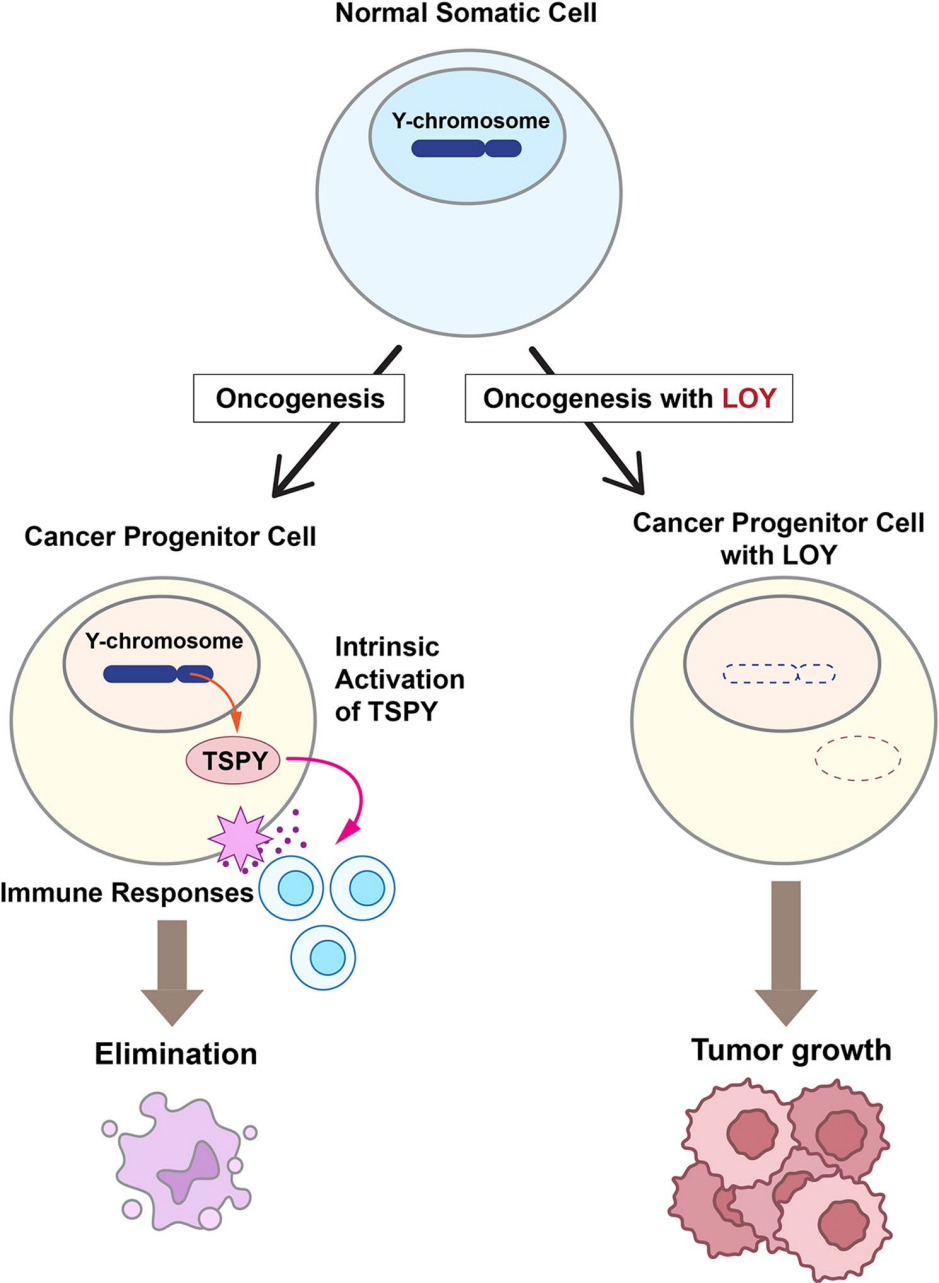
Hypothesis on the contribution of the loss of TSPY gene to cancer predisposition in loss of Y chromosome in men. As a male-specific cancer-testis antigen, TSPY is a guardian gene on the Y chromosome. Its high copy-number of functional units are intrinsically activated in cancer progenitors at early oncogenic stage, eliciting robust humoral and cellular immune responses, thereby eliminating the TSPY-positive cancer progenitor cells and suppressing further oncogenic development (left column). Men with loss of Y chromosome (LOY) lack TSPY expression and the immune responses against the early cancer cells, thereby providing a fostering environment for tumorigenic growth and cancer development (right column)

TSPY is a cancer-testis antigen, normally expressed in the germ cells in the seminiferous tubules of the immune-privileged testis. Its aberrant expression in somatic cancers suggests that it could be subjected to cancer immunosurveillance [14]. Indeed, our recent study clearly demonstrated that TSPY protein possesses extreme immunogenicity, provoking robust humoral and cellular immune responses from the host, thereby eliminating positive tumor cells in a preclinical mouse HCC model [39]. Hence, TSPY could serve dual functions in tumorigenesis: as a tumor suppressor in the early stages and a pro-oncogenic factor in late stages. Its high copy number of protein-coding units [11] insures its expression at the early stages of oncogenesis. Men with low copy-number, i.e. < 20, of TSPY gene on their Y chromosome showed increased susceptibility to prostate cancer [40]. Significantly, tandemly integrated human TSPY transgenes on the mouse Y chromosome could be aberrantly activated during prostatic oncogenesis in a mouse prostate cancer model, suggesting that multiple copies of TSPY genes could be epigenetically and aberrantly activated from the Y chromosome under oncogenic conditions [41]. TSPY could be a guardian gene on the human Y chromosome against cancers. It could be intrinsically activated during early stages of oncogenesis, thereby eliminating positive cancer progenitor cells via immunosurveillance activities and preempting further oncogenesis and cancer development. Currently, it is uncertain if the tandem genomic arrangement and potential ordered spacing of specific transcription factors/chromatin modulators along the highly conserved TSPY units, could contribute to its activation/regulation. However, the high copy-number, i.e. > 42% of all protein-coding genes on the Y chromosome [11], could be adequate for an intrinsic TSPY activation and expression at the time of oncogenesis. Loss of the entire Y chromosome leads to the loss of the TSPY gene cluster, and the ability to activate CD8+ cytotoxic T cells [7, 9], and other immunosurveillance activities, Fig. 1. If such scenario is true, the extent of TSPY positive tumors could be more than those currently identified, since the positivity was assessed at the time of tumor sampling and the TSPY-positive progenitor cells would have been eliminated at the early stage, and therefore would not be detected in tissue samplings at the late stages. As we demonstrated, tumor cells could escape the immune responses through silencing/eliminating of the TSPY gene and/or immune dysfunctions/exhaustion at later stages of oncogenesis [39], at which time the TSPY actions on cell proliferation could exacerbate oncogenic progression. As most TSPY-positive cancer specimens (Table 1) were likely procured at late stages of respective cancers and thus correlated with poor prognosis and survival of the patients [23, 24, 33, 35, 39]. It is reasonable to assume that other genes on the human Y chromosome, particularly those affecting T cell functions [7, 8], could be involved in male-specific cancer immunosurveillance. Recent studies have demonstrated that LOY in T cells could alter transcriptional activities favoring immunosuppression in the tumor microenvironment; and concurrent LOY in tumor cells and T cells exerts worse cancer prognosis and increased cancer mortality in the patients [7]. The loss of the high copy-number and intrinsic oncogenic activation of TSPY in early tumor/progenitor cells with the loss of the entire Y chromosome could play a significant role in contributing to the overall cancer susceptibility/predisposition and poor outcomes for men.

## Supplementary Information

Below is the link to the electronic supplementary material.

Supplementary Material 1.

## Data Availability

No datasets were generated or analysed during the current study.
